# Brandt’s vole hole detection and counting method based on deep learning and unmanned aircraft system

**DOI:** 10.3389/fpls.2024.1290845

**Published:** 2024-03-07

**Authors:** Wei Wu, Shengping Liu, Xiaochun Zhong, Xiaohui Liu, Dawei Wang, Kejian Lin

**Affiliations:** ^1^ Key Laboratory of Agricultural Blockchain Application, Ministry of Agriculture and Rural Affairs & Agricultural Information Institute, Chinese Academy of Agricultural Sciences, Beijing, China; ^2^ State Key Laboratory for Biology of Plant Diseases and Insect Pests, Institute of Plant Protection, Chinese Academy of Agricultural Sciences, Beijing, China; ^3^ Institute of Grassland Research, Chinese Academy of Agricultural Sciences, Key Laboratory of Biohazard Monitoring and Green Prevention and Control in Artificial Grassland, Ministry of Agriculture and Rural Affairs, Hohhot, China; ^4^ Western Agricultural Research Center, Chinese Academy of Agricultural Sciences, Changji, China

**Keywords:** pest rodent monitoring, vole hole detection, unmanned aerial vehicles, deep learning, threshold optimization

## Abstract

Rodents are essential to the balance of the grassland ecosystem, but their population outbreak can cause major economic and ecological damage. Rodent monitoring is crucial for its scientific management, but traditional methods heavily depend on manual labor and are difficult to be carried out on a large scale. In this study, we used UAS to collect high–resolution RGB images of steppes in Inner Mongolia, China in the spring, and used various object detection algorithms to identify the holes of Brandt’s vole (*Lasiopodomys brandtii*). Optimizing the model by adjusting evaluation metrics, specifically, replacing classification strategy metrics such as precision, recall, and F1 score with regression strategy-related metrics FPPI, MR, and MAPE to determine the optimal threshold parameters for IOU and confidence. Then, we mapped the distribution of vole holes in the study area using position data derived from the optimized model. Results showed that the best resolution of UAS acquisition was 0.4 cm pixel^–1^, and the improved labeling method improved the detection accuracy of the model. The FCOS model had the highest comprehensive evaluation, and an R^2^ of 0.9106, RMSE of 5.5909, and MAPE of 8.27%. The final accuracy of vole hole counting in the stitched orthophoto was 90.20%. Our work has demonstrated that UAS was able to accurately estimate the population of grassland rodents at an appropriate resolution. Given that the population distribution we focus on is important for a wide variety of species, our work illustrates a general remote sensing approach for mapping and monitoring rodent damage across broad landscapes for studies of grassland ecological balance, vegetation conservation, and land management.

## 1 Introduction

Small mammals, particularly burrowing rodents, are known as “ecosystem engineers” due to their positive impacts on grassland ecosystems, such as increasing plant diversity, providing shelter to other small creatures from insects to birds, and serving as the food of predators (Li et al., 2023). Nevertheless, because of their rapid reproductive capacity, the population of some species of small rodents can quickly grow and become a biohazard (Singleton et al., 1999). In Inner Mongolia, grasslands cover an area of 54.4 million hectares, the largest ecological function area with the highest biodiversity in northern China. Rodent damage is one of the most significant biohazards in grasslands, resulting in losses of over 200 million tons per year (Liu, 2022).

Brandt’s vole (*Lasiopodomys brandtii*) is a small herbivore rodent species inhabiting Inner Mongolia’s steppes. Its population density experiences dynamic fluctuations annually, with the maximum density reported to be 1,384 voles per hectare and the burrow area damaging approximately 5,616 hectares of grassland (Liu and Sun, 1993). The soil produced by the burrow digging of these voles forms a lot of heterogeneous vegetation patches, resulting in a 65.7% decrease in the yield of high–quality forage (Su et al., 2013). As such, Brandt’s vole is considered to be one of the main pest rodent species, and is thus controlled every year. Monitoring the population size of this species is essential for its scientific management. However, traditional methods are labor–intensive, such as using visual observation or traps to count voles, or using plugging and opening to count active holes, all of which are time–consuming due to the limitation of quadrat to small scales with 0.25 ~ 1 hectare (Du et al., 2022). Therefore, an efficient and accurate technology for pest rodent monitoring is urgent.

By combining rodent density data with satellite remote sensing, it is possible to predict the potential area damaged by rodent pests on a large scale. Spectral indices, such as normalized difference vegetation index (NDVI) and enhanced vegetation index (EVI), extracted from satellite images are strongly correlated with the abundance of rodents in farmland (Andreo et al., 2019; Chidodo et al., 2020; Dong et al., 2023). However, the low resolution of satellite images makes it difficult to accurately monitor rodent damage. Unmanned aircraft system (UAS) near–ground remote sensing technology can be used to take images with a flexible resolution on a relatively large scale, which can improve the evaluation of pest occurrence or damage. Michez et al. (2016) used UAS to assess cornfield damage by wild pigs (*Sus scrofa*), showing that the UAS is more comprehensive than traditional ground assessment. Yi (2017) used UAS to study the broken landscape of the Qinghai–Tibet Plateau, including the platuea pika (*Ochotona curzoniae*) and the platuea zokor (*Eospalax fontanierii*). Tang et al. (2019) used UAS images to study the pikas’ hole landscape pattern and its influence on the surrounding vegetation coverage. They found that the pikas’ holes have a concentrated distribution pattern, and the pikas’ holes can affect the surrounding vegetation influence within 20 cm. Additionally, it was suggested that the pika outbreak may be caused by grassland degradation, providing an ecological basis for pika management. Zhang et al. (2021) investigated the relationship between pika’s holes and alpine meadow bare patches using UAS. They found a variety of short–term relationships between bare patch change and pika interference and suggested that long–term monitoring research with unmanned aerial vehicle technology is necessary. These studies demonstrate that unmanned aerial vehicles are becoming an essential technology tool in grassland ecological monitoring.

For a large number of images generated by UAS, manual visual interpretation remains labor-intensive and time-consuming. Traditional remote sensing-based object detection approaches combine manual features and shallow machine learning models, generally divided into three main steps: (i) selecting regions of interest (ROI); (ii) extracting local features; (iii) applying supervised classifiers to these features. Common methods include maximum likelihood classification (Wen et al., 2018), object-oriented classification (Sun et al., 2019), and support vector machine (SVM) (Heydari et al., 2020). These methods are limited by various backgrounds in the given dataset and are prone to overfitting with limited robustness (Gu et al., 2016). The emerging development of deep neural networks, especially convolutional neural networks (CNNs), has brought significant paradigm shifts and significantly improved the generalization and robustness of automatic learning and feature extraction using annotated training data (Al-Najjar et al., 2019). It more comprehensively describes the differences between various types of objects. The CNN-based object detection algorithm includes anchor-free and anchor-based models. Anchor-based models include Faster R-CNN (Ren et al., 2015), RetinaNet (Lin et al., 2017), SSD (Liu et al., 2016), YOLO (Redmon and Farhadi, 2018), etc. These models need to adjust the hyperparameter settings of the anchor during the training procedure better to match the size of the objects in the dataset. The anchor-free model is more convenient without such a process, and the representative models include CenterNet (Zhou et al., 2019), FCOS (Tian et al., 2019; Tian et al., 2022), etc.

In recent years, the combination of deep learning algorithms and UAS has been widely used for field monitoring, especially for grassland ecological monitoring. This combination has been used to detect wildlife (Kellenberger et al., 2018; Peng et al., 2020) and livestock population surveys (Soares et al., 2021; Wang et al., 2023b). Additionally, it can be used to survey rodents in grassland such as yellow stepped vole (*Eolagurus luteus*; Sun et al., 2019), great gerbil (*Rhombomys opimus*; Cui et al., 2020), plateau pika (*Ochotona curzoniae*; Zhou et al., 2021), the Levant vole (*Microtus guentheri*; Ezzy et al., 2021), Brandt’s vole (Du et al., 2022). By identifying the rodent holes, researchers can estimate the density of rodents. Furthermore, an overall assessment of rodent infestation can be conducted by taking into account above–ground biomass, grass coverage, and other indicators (Hua et al., 2022).

Previous studies have examined a variety of algorithms for recognizing rodent holes in various settings, yet there has been no emphasis on techniques for practical application, such as methods for determining resolution, manual annotation instructions, means of optimizing model parameters, and the design of survey outcomes. In this study, we examined the use of UAS and DL to detect Brandt’s vole holes in the steppe of Inner Mongolia. We explored the effects of different flight heights, manual labeling manners, eight deep learning models, parameter optimization, and model inference methods on the accuracy of vole hole identification. Our finding provides a technological basis for further developing grassland rodent monitoring methods based on UAS and DL.

## 2 Materials and methods

### 2.1 Overview of the study area

**Figure 1:**
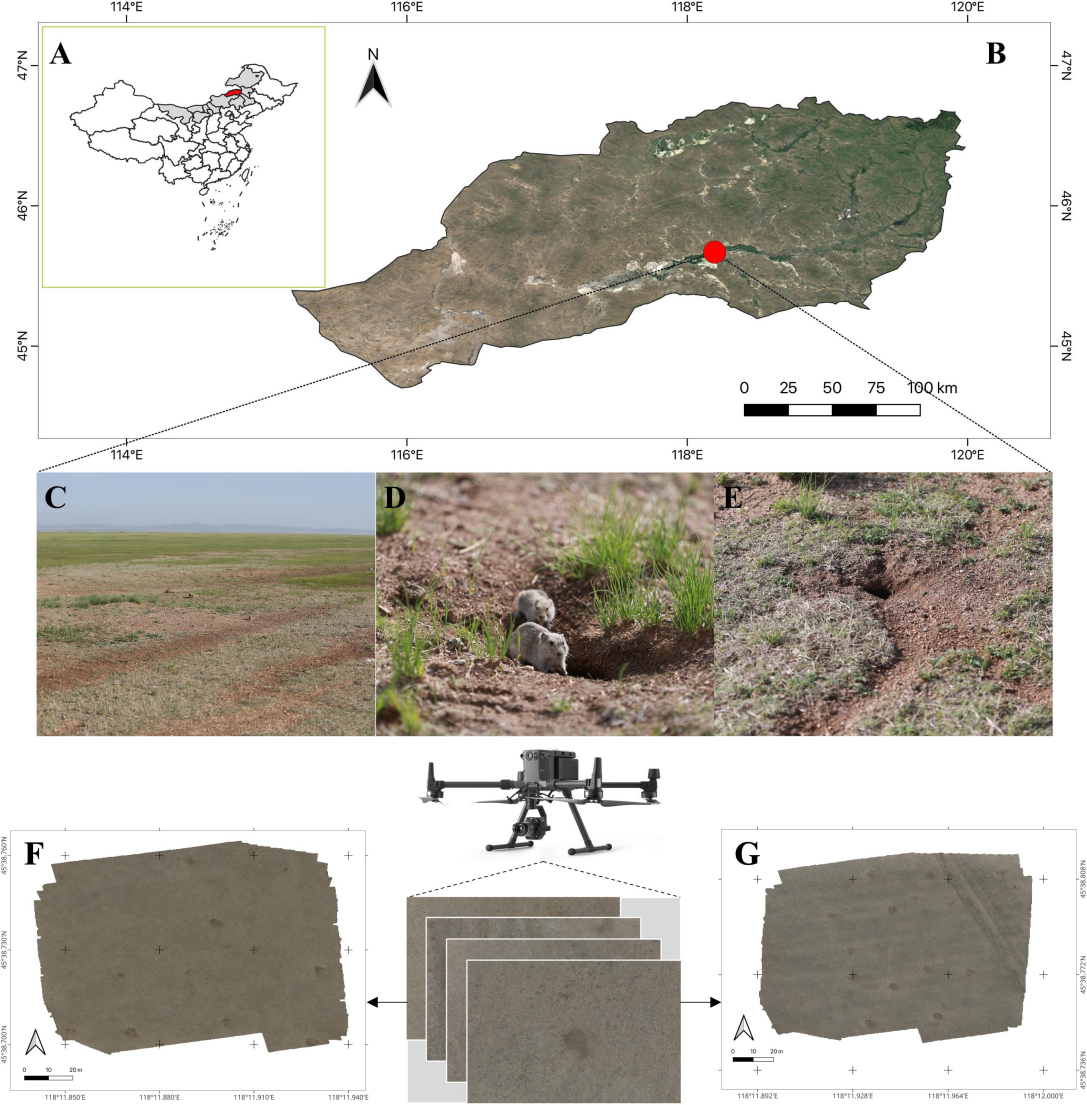
Study area and monitoring sites. **(A)** the position of Inner Mongalia (grey region) and East Ujimqin Banner (red region) in China, respectively; **(B)** the study site in East Ujimqin Banner (red point); **(C)** the burrow area of Brandt’s vole; **(D)** Brandt’s vole; **(E)** vole hole; **(F)** the study area SA1; **(G)** the study area SA2.

The study area is located in the steppe region of Xilingol grassland (118°118’ E, 45°38’ N). It is a high plain terrain with a northern temperate continental climate, an average altitude of 850 m, an average annual temperature of 1.6°C, and an average annual precipitation of 300 mm. The four seasons are distinct in this region. The semi–degraded grassland is dominated by *Stipa krylovii*, *Leymus chinensis*, and *Artemisia frigida*, and the main rodent species is Brandt’s vole (**Figure 1**).

### 2.2 Image acquisition

This study was performed in an area concentrated on Brandt’s vole. Image acquisition was conducted using the DJI M300 RTK (SZ DJI Technology Co., Ltd., Shenzhen, China) equipped with a P1 camera (effective pixel 45 million, 35mm, f2.8), resulting in 8192*5460 pixel images. Prior to commencing formal image acquisition, a 20*30 = 600 m^2^ area (named SA0) was chosen and white lime markers were placed at the four corner points, in order to determine the optimal image resolution for manual visual interpretation. The number of vole holes in the area was manually counted and was recorded as Ground Truth (GT). UAS images of SA0 were obtained from six different flight heights: 10 m, 20 m, 30 m, 50 m, 80 m, and 100 m, resulting in a total of 21 images that constituted a Database. The SA0 area in the images acquired at different flight heights was first segmented in the image processing. The number of vole holes was visually counted, and the result was recorded as Image Truth (IT). Finally, the optimal flight height and corresponding image resolution were calculated by analyzing the relationship between GT and IT. Subsequently, formal image acquisition of the vole holes was conducted based on these findings.

Two different study areas, SA1 and SA2 (**Figures 1F, G**), were selected for image acquisition. The flight route was designed in an S–shape pattern, with a flight height of 30 m, an equidistant photography mode, and a speed of 2 m/s. The overlap rates of waypoints and routes were set to 80% and 70%, respectively. The data gathered from SA1 were named as Data–SA1, consisting of 343 original images, and were used to build and validate a deep learning model. Data–SA2, made up of 234 original images, was acquired from SA2 for testing the application method. An orthophoto image of SA2, measuring 37202*36924 pixels, was created using Agisoft Metashape software (Agisoft LCC., St. Petersburg, Russia). A summary of the data is provided in **Table 1**.

**Table 1:** Image acquisition data information.

Data type	Database	Data–SA1	Data–SA2
**Date of acquisition**	2022–04–07	2022–04–10	2022–04–13
**Weather**	Cloudy	Cloudy	Sunny
**Flight height (m)**	10–20–30–50–80–100	30	30
**Flight mode**	Single point sampling	Route planning	Route planning
**Number of images**	21	343	234
**Validation data**	IT&GT	IT	GT
**Task**	Flight height test	Model building	Application method test

### 2.3 Dataset construction

Due to the large size of the original images in Data–SA1, manual labeling and model training were not feasible. To address this, we divided each original image into 25 sub–images of 1708*1160 in size, resulting in a total of 8575 sub–images. To ensure the accuracy of the truth labels, a rodent pest specialist manually identified the location of vole holes in each sub–image and labeled them using Labelme 4.5.10 (https://github.com/wkentaro/labelme) software. After removing images without vole holes, 6894 valid images were left with 24564 vole holes marked. The training, validation, and test sets were divided in a 5:2:3 ratio, resulting in 3447, 1379, and 2068 images, respectively. **Supplementary Figure 1** illustrates the image segmentation and manual visual interpretation labeling process.

### 2.4 Deep learning algorithm

**Figure 2:**
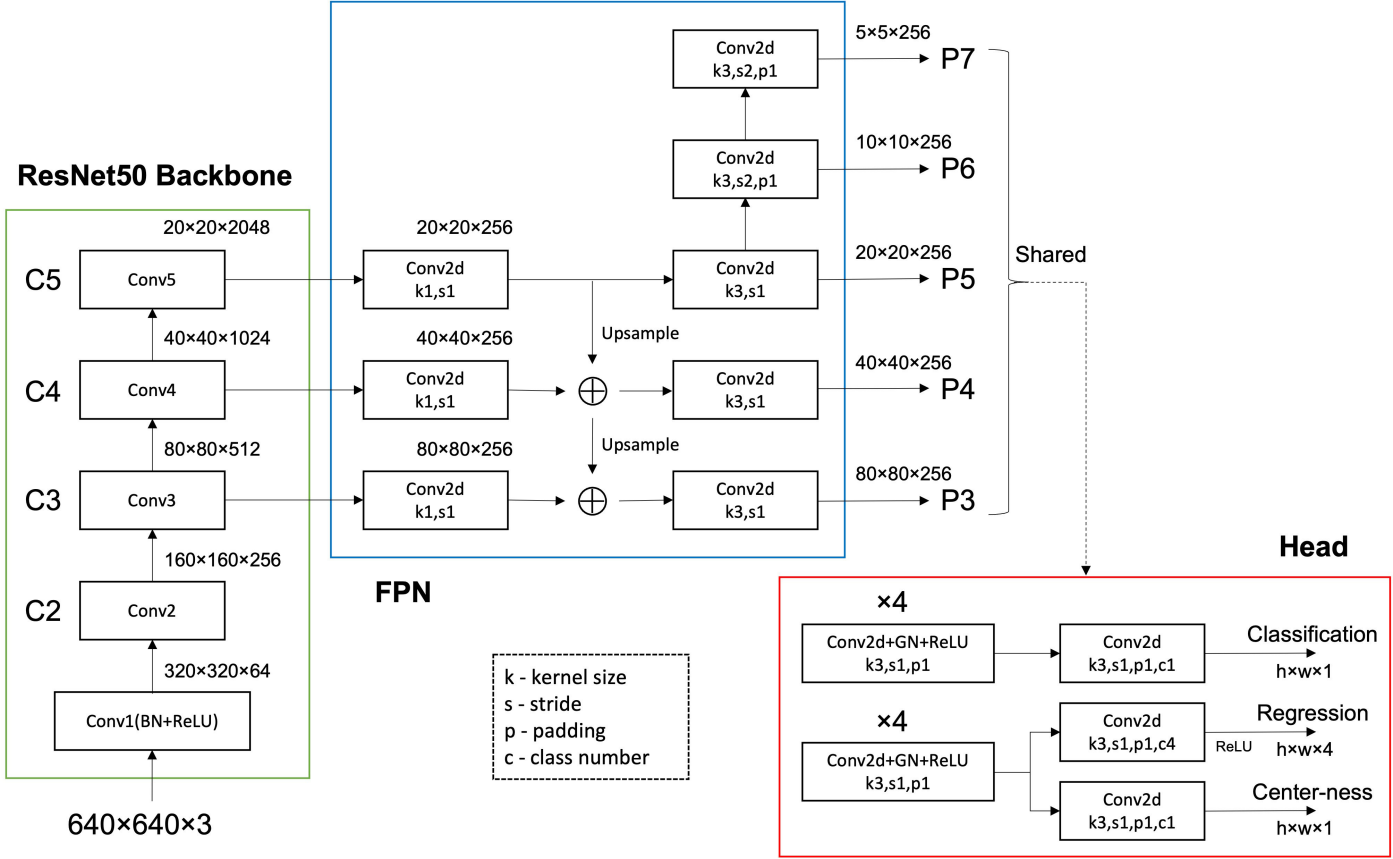
The structure of the FCOS network.

Traditional anchor-based detection models often perform poorly with small targets due to low match rates between the small targets and anchor boxes. The FCOS (Tian et al., 2019; Tian et al., 2022) model adopts an anchor-free design, allowing direct object localization and classification on feature maps, effectively solving the problem of small object detection. Additionally, FCOS excels in reducing false positives, thanks to its unique center-ness scoring mechanism. This mechanism evaluates the closeness of each predicted box’s center to the actual target center, effectively distinguishing real targets from background noise. This is crucial in distinguishing positive and negative samples, especially in small target detection tasks. The detection of vole holes in our dataset is a standard small target detection task, making this anchor-free algorithm more suitable. To determine the most effective approach for investigating vole holes, we compared several deep learning algorithms, including Faster–rcnn (Ren et al., 2015), SSD (Liu et al., 2016), and five YOLO series algorithms (Redmon and Farhadi, 2018; Bochkovskiy et al., 2020; Ge et al., 2021; Zhu et al., 2021; Wang et al., 2023a). **Figure 2** shows the structure of the FCOS network. The Backbone module generates P3, P4, and P5 from the C3, C4, and C5 outputs. P6 is produced from a 3×3 kernel convolutional layer with a stride of 2. Based on P6, P7 is created using a 3×3 kernel convolutional layer with a stride of 2. Finally, the network utilizes a shared Head detector for P3 to P7, containing Classification, Regression, and Center–ness.

For the Classification, n score parameters are predicted at each position of the predicted feature map, with the number of categories being 1. For the Regression, four distance parameters are predicted at each position in the predicted feature map, which are the distances to the left, top, right, and bottom of the object (1, t, r, and b, respectively). For the Center–ness, one parameter is predicted at each position of the predicted feature map, which reflects the distance of the point from the object center, with its value domain being between 0 and 1. The closer to the object center, the higher the center–ness value. The loss function is composed of classification loss, location loss, and center–ness loss. The equation (Equation 1) is as follows:


(1) $$\begin{array}{l} L\left(\left\{p_{x,y}\right\},\left\{t_{x,y}\right\},\left\{s_{x,y}\right\}\right)=\frac{1}{N_{pos}}\sum_{x,y}L_{cls}\left(p_{x,y},c_{x,y}^{*}\right)\\ +\frac{1}{N_{pos}}\sum_{x,y}1_{\left\{c_{x,y}^{*}>0\right\}}L_{reg}\left(t_{x,y},t_{x,y}^{*}\right)\\ +\frac{1}{N_{pos}}\sum_{x,y}1_{\left\{c_{x,y}^{*}>0\right\}}L_{ctrnes}\left(s_{x,y},s_{x,y}^{*}\right) \end{array}$$

where $L_{cls}$ is focal loss as in (Lin et al., 2017) and $L_{reg}$ is the GIOU loss (Tian et al., 2020). $N_{pos}$ denotes the number of positive samples and λ being 1 in this paper is the balance weight for $L_{reg}$ . $p_{x,y}$ represents the predicted scores of each (x,y) at the feature map; $C_{x,y}^{*}$ represents the true label of each (x,y) at the feature map; $1_{\left\{c_{x,y}^{*}>0\right\}}$ is equal to 1 when the point (x,y) is matched to a positive sample and 0 otherwise; $t_{x,y}$ indicates the information of predicted bounding box of (x,y) at the feature map, while the $t_{x,y}^{*}$ indicates the true information; $s_{x,y}$ indicates the predicted center–ness of (x,y) at the feature map, and the $s_{x,y}^{*}$ indicates the true center–ness.

All models were trained on Ubuntu 20.04.1 LTS with Python 3.7, PyTorch 1.7.1, and CUDA 11.0. The server was equipped with a GPU–A100, a CPU–AMD EPYC 7742, and 512 GB of RAM. The hyperparameters for all models are listed in **Supplementary Table 1**. Additionally, Mosaic data augmentation (Bochkovskiy et al., 2020) was used during model training.

### 2.5 Evaluation indicators

To understand the practical application of UAS and deep learning algorithms in the survey of vole holes, we evaluated four perspectives: unmanned aerial vehicle flight heights, model accuracy, model optimization, and the verification of regional vole hole numbers. The evaluation methods used were as follows.

#### 2.5.1 Resolution evaluation

The goal of resolution evaluation is to identify the ideal image resolution for regional surveys of Brandt’s vole holes. Based on the GT and IT in the same area, using True Positive (TP), False Positive (FP), False Negative (FN), Precision, Recall, and F1 score to evaluate the performance of manual visual interpretation. The Precision, Recall, and F1 score are calculated based on TP, FP, and FN. The equations are as follows:


(2) $$Precision=\frac{TP}{FP+TP}$$

(3) $$Recall=\frac{TP}{FN+TP}$$

(4) $$F1\;score=\frac{2Precision*Recall}{Precision+Recall}$$

#### 2.5.2 Model accuracy evaluation

In the model evaluation, we use Precision, Recall, and F1 score to measure the performance of each model. However, in contrast to the previous approach, the definitions of TP, FP, and FN are based on the concept of intersection over union (IoU). This is the ratio between the overlapping area of the detection bounding box and labeling bounding box and the formed area of two bounding boxes. The selection of the threshold can determine these three indexes, and the threshold is usually set to 0.5. The equations are given in Equations 2–4.

The mean Average Precision (mAP) is utilized to evaluate the model, which is equal to the area under the Precision–Recall Curve (PRC). PRC is formed by plotting the precision versus the recall for various confidence levels of the network prediction. It shows the influence of the confidence level on the correlation between recall and precision. The AP calculation is as follows (Equation 5):


(5) $$AP@\alpha =\sum^{}\int_{0}^{1}Precision\left(Recall\right)dRecall$$

where α is the IoU threshold for which precision and recall are determined, and dRecall is the differential of the recall. To calculate the mAP, the average of the APs for each class of the object detection task is taken. Science there was only one class in this study, the AP and mAP were the same.

#### 2.5.3 Model optimization evaluation

Evaluating model accuracy can help choose the best model, but it may not necessarily provide the optimal performance due to certain limitations such as the IoU threshold and confidence threshold. The task of counting vole holes falls under counting regression, whereas the aforementioned indexes are geared towards classification. To further transform the task of counting vole holes towards a counting regression task, we conducted a thorough analysis to identify the optimal IoU threshold and confidence threshold, as well as utilizing more effective evaluation indexes. Specifically, we adopted miss rate (MR) and false positives per image (FPPI) to calculate the average false detection rate of each image. The equations are as follows (Equations 6, 7):


(6) $$FPPI=\frac{FP}{N}$$

(7) $$MR=\frac{FN}{GT}=1-Recall$$

where N is the number of pictures, the FP is the number of false vole holes detected.

To evaluate detectors, we plot the MR against the FPPI (using log–log plots) by varying the threshold on detection confidence. This is more suitable than PRC for our tasks, as there is typically an upper limit on the allowable FPPI rate regardless of object density (Dollar et al., 2012). To summarize detector performance, the log–average miss rate (LAMR) is computed by averaging the MR at nine FPPI rates that are evenly spaced in log space in the range 10^–2^ to 10^0^ (for curves that end before reaching a given FPPI rate, the minimum MR achieved is used).

When selecting a confidence threshold, it is typical to use the F1 score as an evaluation metric. However, the F1 score does not provide a clear indication of how many errors are present in the task. False and missed detections are both negative outcomes that often occur together at a certain confidence level. To ensure the accuracy of the final count, the sum of false and missed detections must be minimized. Thus, the mean absolute percentage error (MAPE) is therefore chosen as the final evaluation metric, and the optimal confidence threshold can be calculated by minimizing the MAPE. The equation is as follows:


(8) $$MAPE=\frac{1}{n}\sum_{i}\frac{\left|ES_{i}-GT_{i}\right|}{GT_{i}}\times 100\%$$

where $ES_{i}$ is the number of model estimate results, $GT_{i}$ is the number of ground truth result; and n is the total number of images.

#### 2.5.4 Verification of the number of regional vole holes

Data–SA2 was selected to validate the regional survey of optimal model for vole holes. To measure the accuracy of the optimized model estimation, the determination coefficient (R^2^), root mean square error (RMSE), and MAPE (Equation 8) were utilized. The equations are as follows (Equations 9–11):


(9) $$R^{2}=1-\frac{\sum_{i}^{n}{\left(x_{i}-y_{i}\right)}^{2}}{\sum_{i}^{n}{\left(x_{i}-\bar{x}\right)}^{2}}$$

(10) $$RMSE=\sqrt{\frac{\sum_{i}{\left(x_{i}-y_{i}\right)}^{2}}{n}}$$

(11) $$Accuracy=1-\frac{\left|N_{ɡt}-N_{est}\right|}{N_{ɡt}}\times 100\%$$

where $x_{i}$ is the model measurement results, $\bar{x}$ is the mean of the model measurement, $y_{i}$ is the manual measurement results (IT); and n is the total number of measurements; $N_{est}$ is the result of model estimation, and $N_{ɡt}$ is the manual survey result.

## 3 Results

### 3.1 Flight height

A suitable flight height was identified by contrasting the differences between GT and IT at distinctive flight heights. **Supplementary Figure 2** shows a clear comparison between vole holes and distractors in high resolution and low resolution. As the flight height increases, **Table 2** reveals a decrease in the number of manual visual interpretations, a reduction in TP, an increase in FP, and a higher probability of missing detection. Even though accuracy values remain the same, recall values are significantly lower. According to the F1 score results, manual visual interpretation performs best when the flight height is not higher than 30 m. To maximize the survey area, a flight height of 30 m is recommended for achieving an F1 score of 0.98 and an image resolution of 0.4 cm/pixel.

**Table 2:** Manual visual interpretation results at different flight heights.

Flight Height (m)	GT	IT	TP	FP	FN	Accuracy	Recall	F1 score	Resolution ratio (cm/pixel)
10	55	55	54	1	0	0.98	0.98	0.98	0.1
20	55	55	54	1	0	0.98	0.98	0.98	0.3
30	55	53	53	2	0	1.00	0.96	0.98	0.4
50	55	54	51	4	3	0.94	0.93	0.94	0.6
80	55	49	46	9	3	0.94	0.84	0.88	1.0
100	55	36	36	19	0	1.00	0.65	0.79	1.3

### 3.2 Model performance

**Figure 3:**
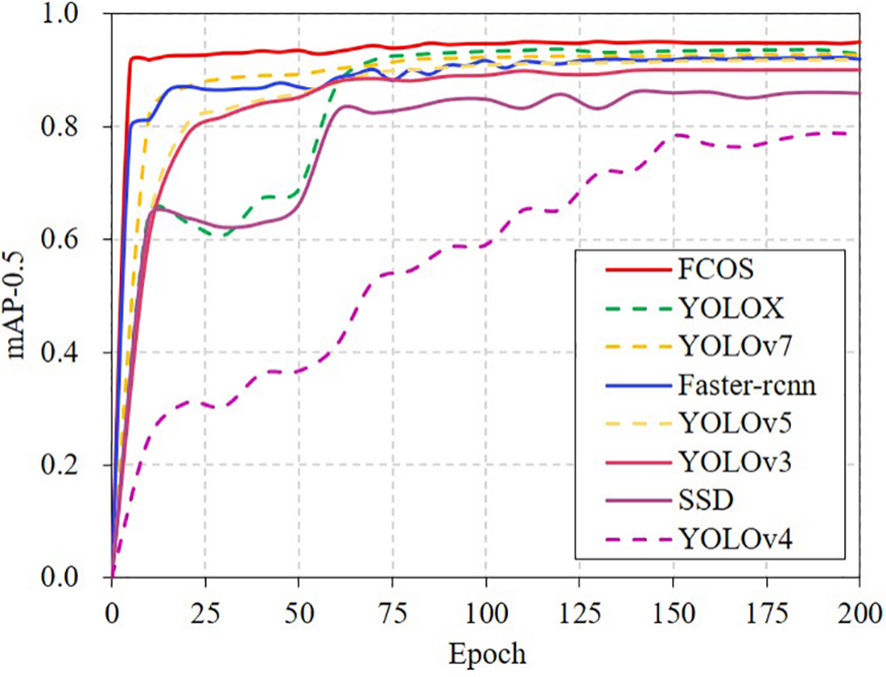
Validation mAP curves for the different models.

To select the best model, eight object detection algorithms were evaluated. The model was constructed using a simultaneous training and validation model, and a pre–trained mode was used for faster convergence. The backbone network weights, which extract generic basis features, were kept the same for the first 50 steps, and the optimization weights were adjusted globally afterwards. The training and validation results are illustrated in **Supplementary Figure 3** and **Figure 3**. After 60 epochs, most models reached their peak mAP values and had stabilized, showing successful model training.

The performance of each model in recognition of vole holes was good (**Supplementary Figure 4**), but the confidence level and position of the detection box varied, which could affect the subsequent vole hole counting results. According to the comprehensive evaluation indexes of the different models (**Table 3**), FCOS had the highest mAP of 95.19%. Faster–rcnn model had the highest Recall value of 95.91%, but its Precision value was only 69.07%, resulting in a higher Recall at the expense of precision compared to other models. The F1 score was used to evaluate model performance by combining both Precision and Recall. FCOS and YOLOX performed the best, and their F1 score values were both above 0.89. Additionally, the model’s size and speed were evaluated. SSD had the highest Frame Per Second (FPS) at 161.08, while Faster–rcnn had the slowest speed with an FPS of 25.71.

**Table 3:** Comprehensive performance of the different models.

Models	mAP	Precision	Recall	F1 score	Latency/s	FPS	Parameters
Faster–rcnn	92.29	69.07	95.91	0.80	0.0389	25.71	28.28 M
SSD	89.67	85.85	82.37	0.84	0.0062	161.08	23.75 M
FCOS	95.19	84.70	93.28	0.89	0.0200	50.12	32.12 M
YOLOv3	89.05	87.25	77.07	0.82	0.0175	57.27	61.52 M
YOLOv4	80.81	86.33	60.09	0.71	0.0261	38.38	63.94 M
YOLOv5	92.98	86.24	85.70	0.86	0.0161	62.29	7.06 M
YOLOX	94.56	88.19	89.79	0.89	0.0173	57.94	8.94 M
YOLOv7	93.52	85.91	89.16	0.88	0.0199	50.36	37.19 M

### 3.3 Model optimization

**Figure 4:**
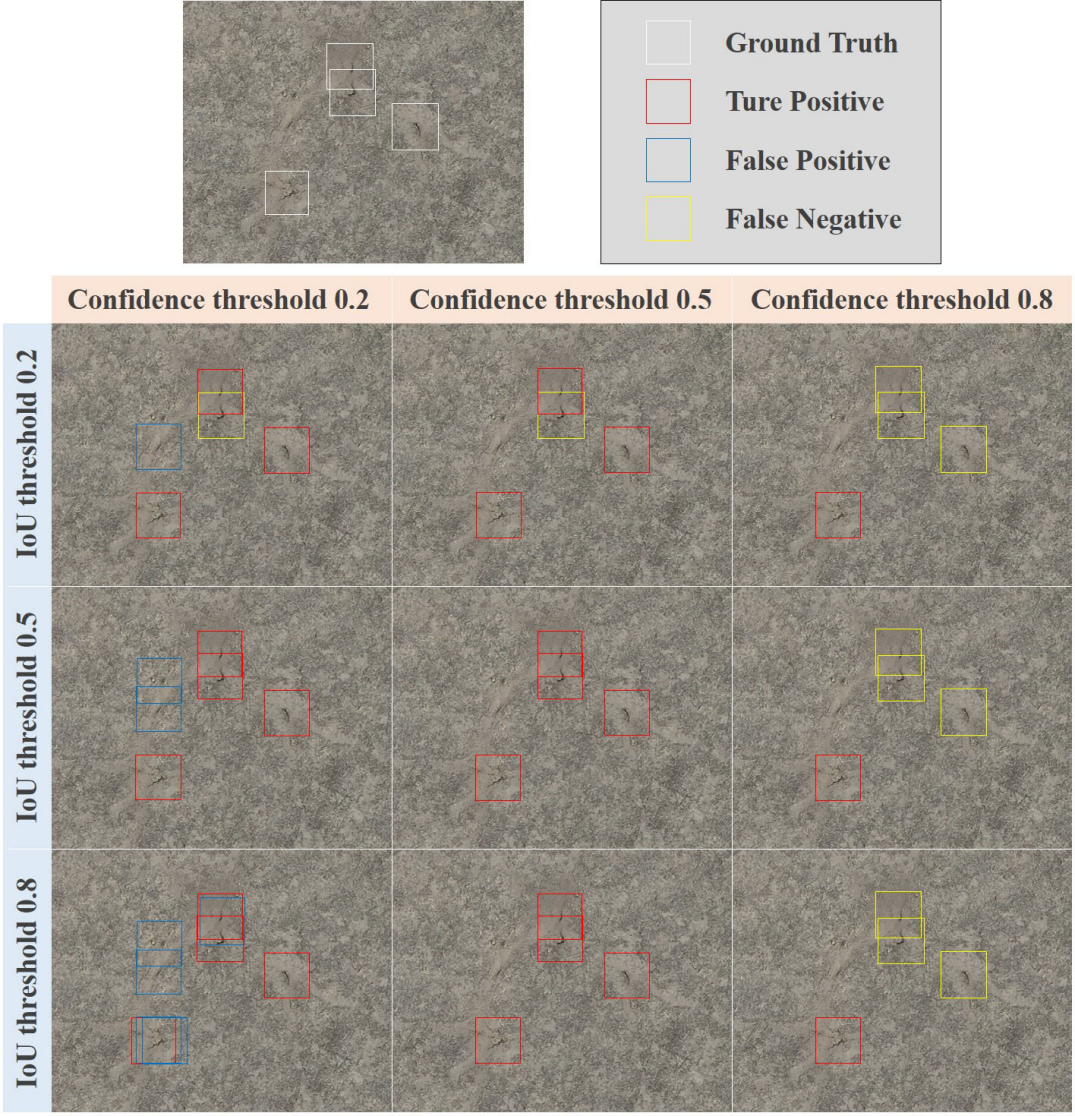
The detection results of different IoU threshold and confidence threshold.

In the model training stage, the objective factors affecting the FP and FN are complex environment (e.g., light, occlusion), similars (e.g., feces) and the model structure. Data augmentation and improved model algorithms are often used to reduce the impact of these factors. In the model inference stage, parameters such as IoU threshold and confidence threshold have a certain impact on the prediction accuracy (**Figure 4**), and these parameters can be artificially adjusted to optimize the prediction results. The aims of this study are to examine the regression performance of the number of vole holes by transforming the object detection task into an estimate regression task performance. To do so, two issues must be addressed: setting the threshold (i.e. the IoU) for determining when the object box and prediction box overlap enough to be considered a positive sample, and selecting the confidence threshold for considering a sample as positive.

#### 3.3.1 IoU threshold

**Figure 5:**
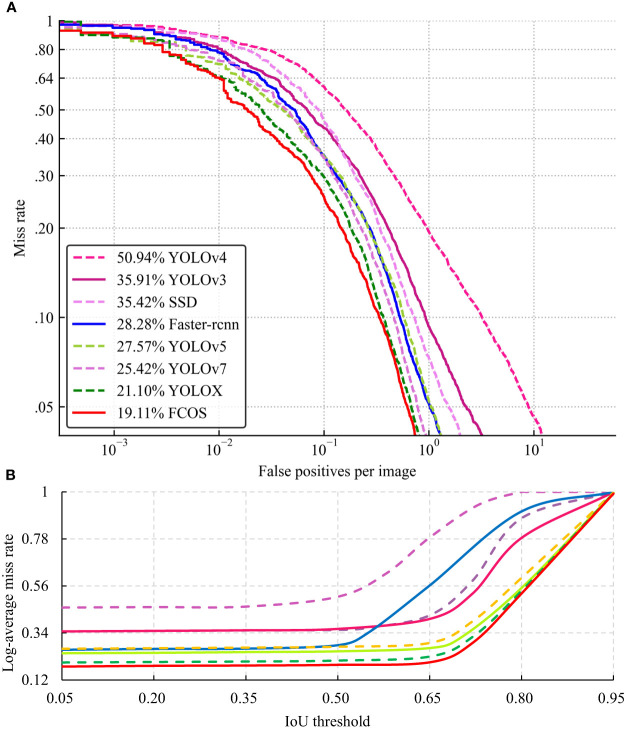
The MR–FPPI curves for the different models **(A)** and LAMR values change at different IoU thresholds **(B)**.

The MR–FPPI curve is a useful tool for the performance of a detector based on the IoU threshold. **Figure 5A** shows the MR–FPPI curves for IoU values below 0.5, with FCOS having the best performance and a LAMR value of 19.11%. **Figure 5B** illustrates the LAMR performance of all models at 7 IoU thresholds, including 0.05, 0.20, 0.35, 0.50, 0.65, 0.80, and 0.90. Each model has an inflection point above which the LAMR value increases rapidly, while the value tends to level off otherwise. As the LAMR value decreases, the detector performance improves, and as the IoU threshold increases, the detection box becomes more accurate. Therefore, the inflection point indicates the optimal IoU threshold for each model. Consequently, the best IoU threshold for Faster–rcnn and YOLOv4 was 0.50, while for the other models, it was 0.65.

#### 3.3.2 Confidence threshold

**Figure 6:**
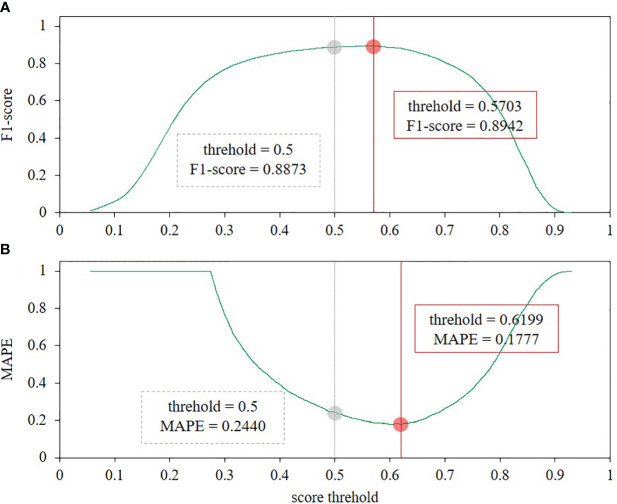
Performance of the FCOS model at different confidence thresholds. **(A)** the F1 score indicator, and **(B)** the MAPE indicator.

As shown in **Figure 6**, using the FCOS model as an example, the gray dashed line and the red solid line represent its confidence performance at the 0.5 standard level and when the highest F1 score or the lowest MAPE value. These two indicators do not correspond to the same confidence level. However, MAPE is more consistent with counting regression, so it is used instead of F1 score. **Table 4** reveals that all models have better values of F1 score and MAPE at their best confidence level than the 0.5 level. The best confidence levels obtained for both F1 and MAPE metrics were also different for all models. In particular, FCOS achieves the best result with a confidence threshold of 0.62 and an MAPE of 0.1777.

**Table 4:** Performance of the different models at different confidence thresholds.

	Threshold = 0.5		Optimized Confidence Threshold			
Models	F1 score	MAPE	F1 score	Threshold	MAPE	Threshold
Faster–rcnn	0.8031	0.5518	0.8666	0.91	0.2181	0.96
SSD	0.8407	0.2532	0.8477	0.38	0.2422	0.55
FCOS	0.8879	0.2427	0.8942	0.57	0.1777	0.62
YOLOv3	0.8185	0.2653	0.8312	0.34	0.2604	0.47
YOLOv4	0.7086	0.366	0.7635	0.32	0.3124	0.38
YOLOv5	0.8597	0.2386	0.8604	0.47	0.2268	0.58
YOLOX	0.8898	0.2111	0.8900	0.51	0.2010	0.62
YOLOv7	0.8751	0.2313	0.8759	0.52	0.2147	0.59

### 3.4 Model inference

**Figure 7:**
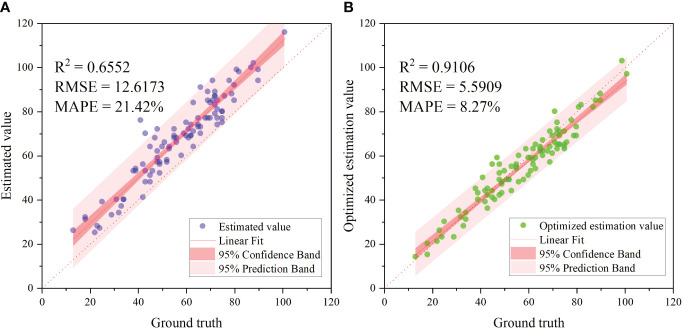
Validation of the model counting results. **(A)** is the model detection result before optimization, and **(B)** is after optimization.

**Figure 8:**
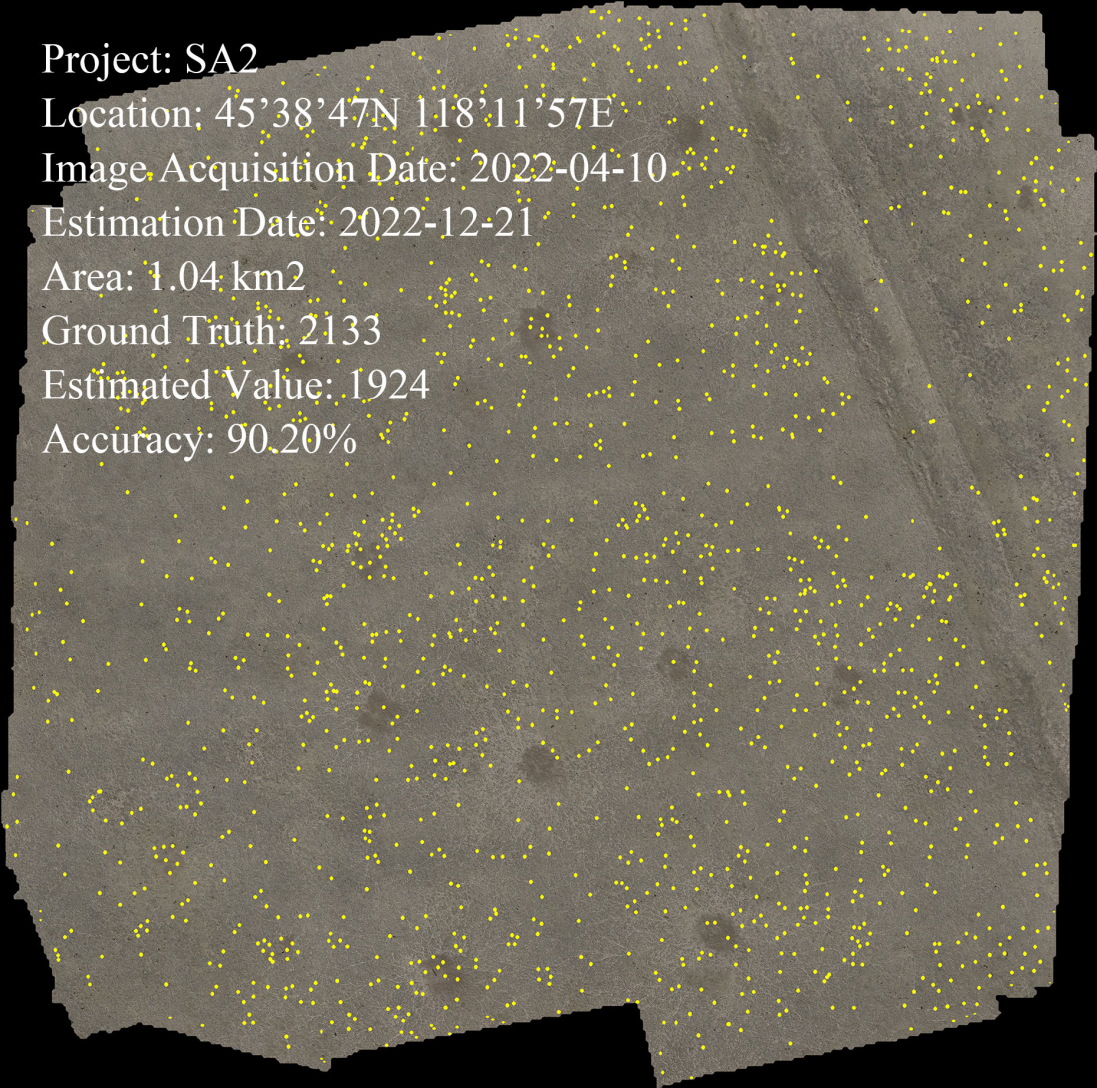
Results of region vole holes counting.

We chose the FCOS model as the best model for further exploring the quantity of vole holes. The validation data was the original images from Data–SA2 and the model was evaluated using an IoU threshold of 0.5 and a confidence threshold of 0.5 before optimization, and an IoU threshold of 0.65 and a confidence threshold of 0.62 after optimization. The model was compared to GT and the results are shown in **Figure 7**. Before optimization, the R^2^ was 0.6552, the RMSE was 12.6173, and the MAPE was 21.42%. The high false detection rate led to an unsatisfactory result. After optimization, the performance of the model was greatly improved with an R^2^ of 0.9106, RMSE of 5.5909, and MAPE of 8.27%. The threshold parameters were adjusted using the new assessment indexes to balance the number of false detections and the number of missed detections. Thus, the optimized model can be used as an effective method to detect the number of Brand’s vole holes. The accuracy of vole hole counting of the stitched orthophoto was 90.20% (**Figure 8**).

## 4 Discussion

### 4.1 Comparison of different labeling methods

During object labeling, it is typical to draw a rectangular box to mark the object boundary (Label–1, **Supplementary Figure 5A**). However, the cow dung (**Supplementary Figure 5B**) in UAS images is similar to the vole holes, making it difficult to simply label the vole hole boundary due to the presence of distractors. We found the vole trails always exist around the holes, which isa distinct feature of vole–damaged vegetation. Hence, we enlarged the box range for labeling (Label–2, **Supplementary Figure 5C**).

**Figure 9:**
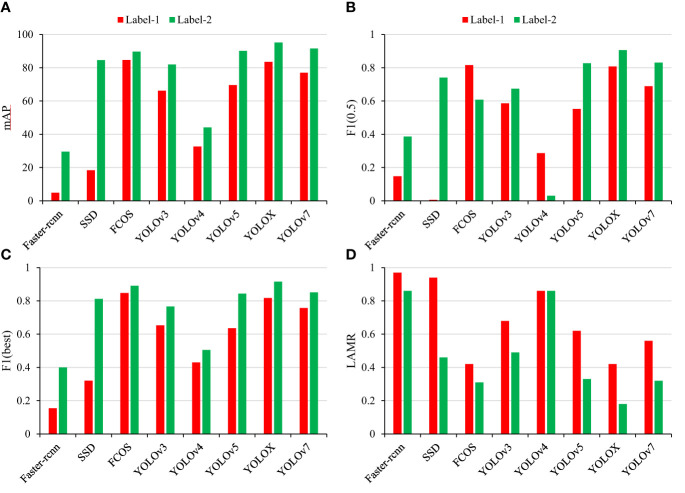
Comparison of the results of different models using two labeling methods. **(A)** mAP, **(B)** F1 score–0.5, **(C)** F1 score–best, and **(D)** LAMR.

To assess the effectiveness of the proposed labeling technique, 1125 sub–images were randomly chosen from Data–SA1 and labeled using both Label–1 and Label–2 methods. These images were then divided into training, validation, and test set in a 5:2:3 ratio. The results (**Figure 9A**) demonstrate that the mAP values of all models trained with Label–2 are higher than those of Label–1. However, this pattern is not obvious when the confidence threshold is set to 0.5 (**Figure 9B**). Nevertheless, when the confidence threshold is optimized, the F1 score and LAMR values of the models follow the aforementioned pattern (**Figures 9C, D**). Therefore, the improved Label–2 labeling method offers clear advantages over the traditional Label–1 method.

### 4.2 The challenge of repeat counting

**Figure 10:**
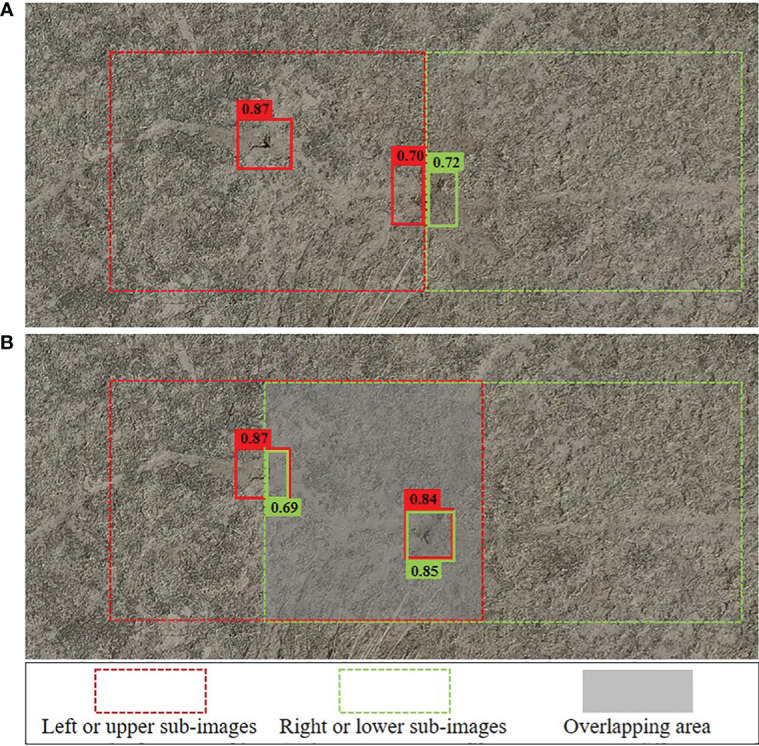
Repeat counting problem, **(A)** no overlapping area, **(B)** with overlapping area.

When a hole is located at the edge of a sub–image segmentation (**Figure 10A**) during model inference, it may be misidentified as two separate holes, resulting in a decrease in the confidence of both holes. This can lead to an inaccurate counting result, regardless of whether the confidence is higher or lower than the threshold we set. To address this issue, we designed an overlapping area between adjacent sub–images (**Figure 10B**) to form a situation of multiple detections for one hole. We then used a redundancy removal algorithm, Non–Maximum Suppression (NMS), to eliminate redundant detection boxes. NMS is commonly used to eliminate redundant data by setting an IoU threshold. However, the IoU threshold in overlapping areas can be either small or large, making it difficult to select the right threshold, especially for two vole holes that are close together. To address this challenge, we improved the NMS by introducing the intersection over a smaller (IoS) metric, which is the ratio of the intersection to the smaller bounding box (**Supplementary Figure 6**, Equation 12). This approach ensures that the IoS values of the repeated bounding boxes are close to 1, while the IoS values of the two close vole holes’ bounding boxes remain small. The IoS threshold can also be easily chosen (the IoS threshold in this study was 0.5). This method with overlapping area processing decreased the error rate by 1.03% (**Table 5**). The width (or height) of the overlapping area was usually 2–3 times the size of the vole hole bounding box. Too much overlapping would increase the calculation amount and reduce efficiency, while too little overlapping would not work.

**Table 5:** Effect of overlapping area on repeat counting.

Methods	R^2^	RMSE	MAPE
Non–overlapping area	0.8804	6.5661	9.31
Overlapping area	0.9106	5.5909	8.27


(12) $$IoS=\frac{A\;\cap \;B}{B}$$

### 4.3 Image resolution and model optimization

Despite the varying required image resolution among different rodent species (Cui et al., 2020; Ezzy et al., 2021; Zhou et al., 2021; Du et al., 2022), there has been no research on the method of determining the UAV flight altitude or image resolution. This study uses manual visual interpretation as a benchmark and conventional classification evaluation indexes for resolution evaluation. This approach can determine the best image resolution which balances the accuracy and efficiency. Previous studies have mainly concentrated on refining model structure, while disregarding model application techniques. Even though model accuracy can be improved, incorrect application can lead to more accuracy loss. Therefore, this study transforms the object detection results into regression count results to optimize the application stage of model through more suitable evaluation metrics, thus allowing the full utilization of the capabilities of a mature model.

## 5 Conclusion

In this study, Brand’s vole hole counting was used as an example to explore the complete technical route of rodent hole counting, which promoted the application of UAS and DL in grassland rodent damage monitoring. We determined the optimal image resolution suitable for UAS monitoring, improved the conventional vole hole labeling method, selected the FCOS algorithm with anchor-free design as the rodent hole detection model, and adopted the regression strategy for the first time to optimize the model inference process. The results showed that the image resolution is most suitable when the flight altitude is 30 m and the mAP of FCOS model reached 95.19%. Compared with the GT, the accuracy of the optimized model could reach 90.20%. The above results indicate that our method is an effective and efficient method for detecting rodent holes in grassland. However, due to the constant seasonal and inter-species changes in the morphology of vole holes and their surrounding vegetation, it is currently difficult to develop a universal extraction algorithm. The development of large-scale grassland ecological monitoring model will be an important research topic in the future. Nonetheless, the ever–changing morphology of rodent holes and the surrounding vegetation across seasonal and inter–species, make it difficult to develop a universal extraction algorithm at present. It is expected that the development of large models for grassland ecological monitoring will be a key research topic in the near future.

## Data availability statement

The data presented in the study are deposited in the Github repository, and can be found here: https://github.com/Weiwu327/Brandt-s-vole-hole-detection-and-counting-method-based-on-deep-learning-and-unmanned-aircraft-system.git.

## Author contributions

WW: Conceptualization, Formal analysis, Methodology, Software, Visualization, Writing – original draft. SL: Funding acquisition, Resources, Writing – review & editing. XZ: Resources, Supervision, Writing – original draft. XL: Data curation, Investigation, Writing – original draft. DW: Conceptualization, Funding acquisition, Supervision, Validation, Writing – original draft, Writing – review & editing. KL: Project administration, Resources, Writing – review & editing.
