# Supplementary material for: Brandt’s vole hole detection and counting method based on deep learning and unmanned aircraft system
Source: Front Plant Sci. 2024 Mar 7;15:1290845. doi: 10.3389/fpls.2024.1290845 (PMC10955068; doi:10.3389/fpls.2024.1290845)
Supplement: Supplementary file 1 [file DataSheet_1.docx]

Supplementary Material

# Supplementary Figures


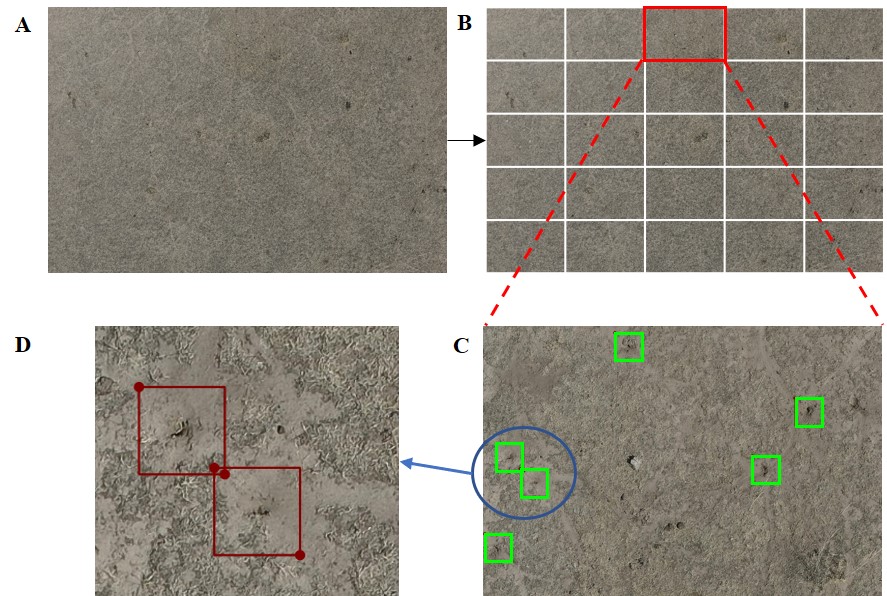
**Supplementary Figure 1.** A schematic diagram of image segmentation and manual visual interpretation labeling. (A) the original image, (B) sub–images, (C) sub–image labeling, and (D) an enlarged view of the vole hole labels.


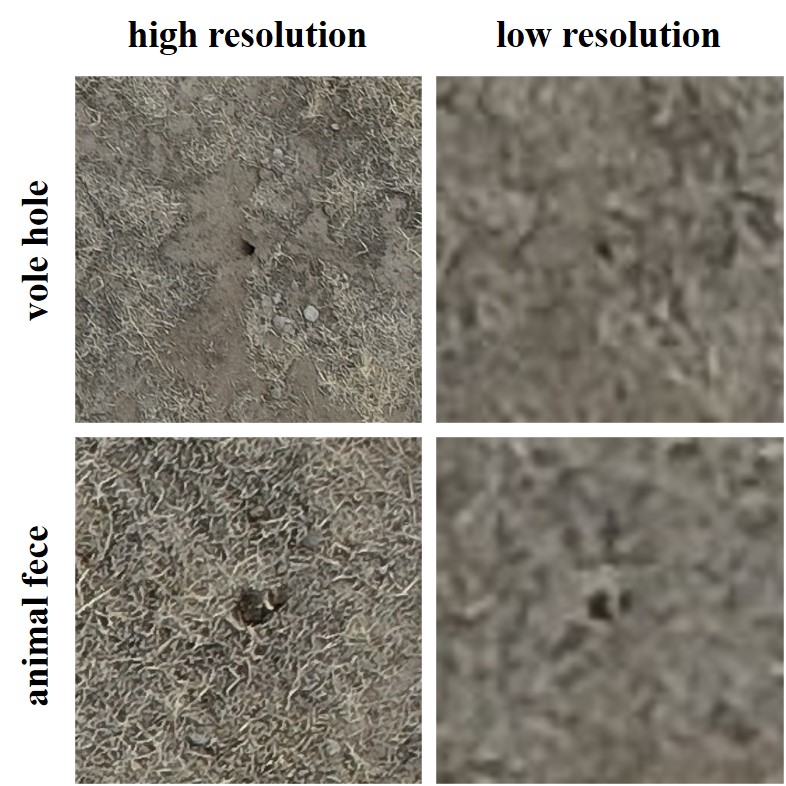


**Supplementary Figure 2.** Image of vole holes and animal feces taken from different resolutions.


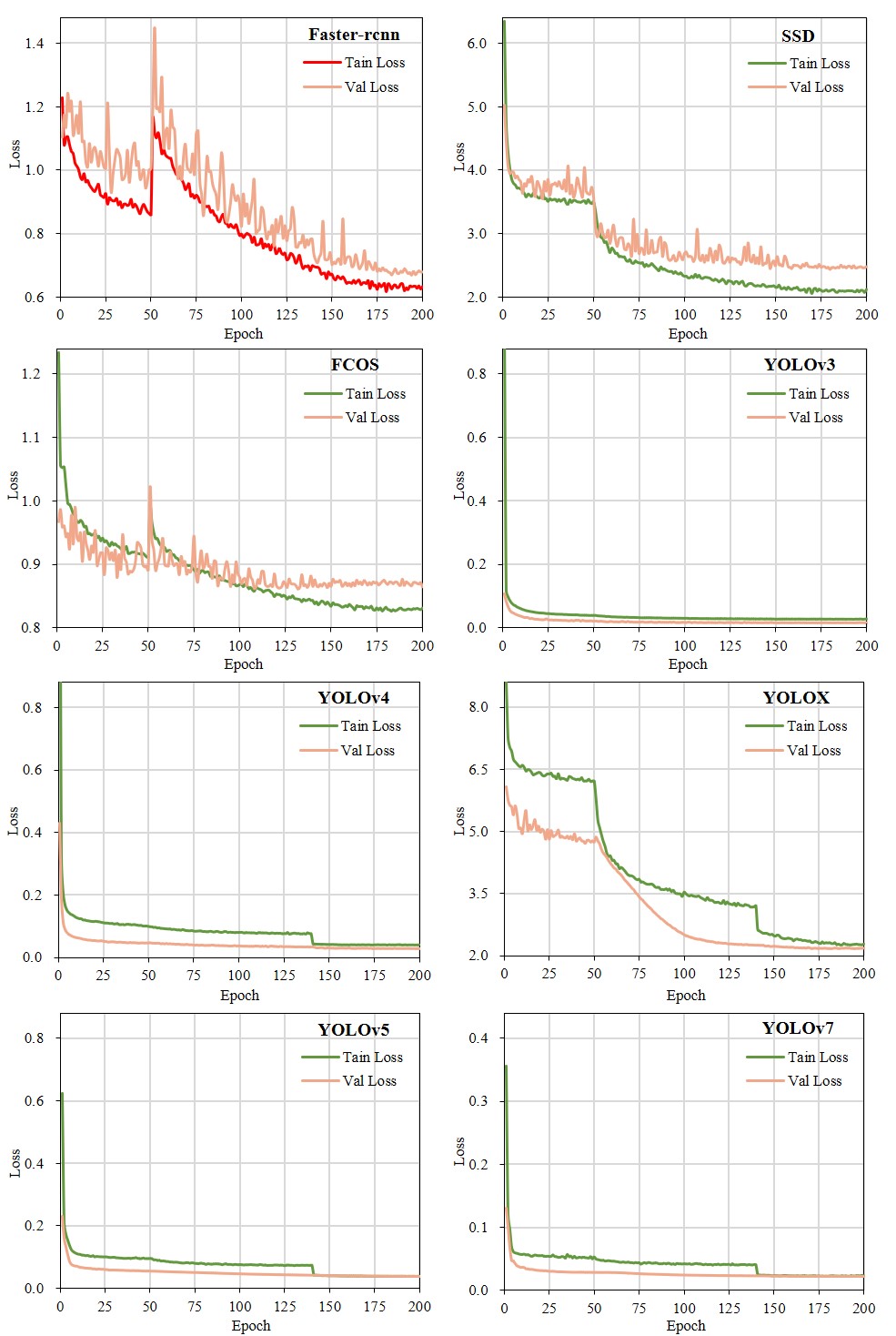


**Supplementary Figure 3.** Training and validation loss curves for the different models.


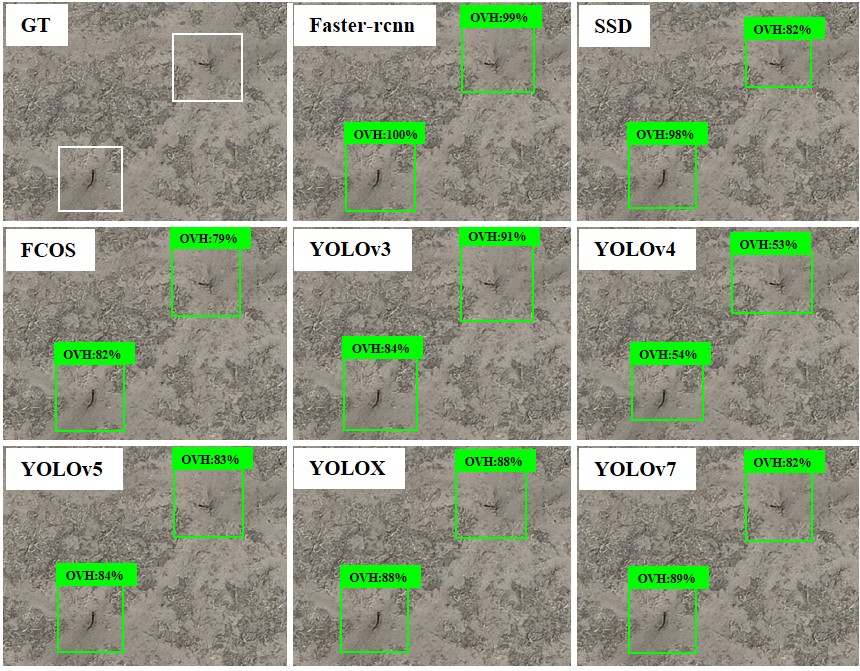


**Supplementary Figure 4.** Identification effects of the different models.


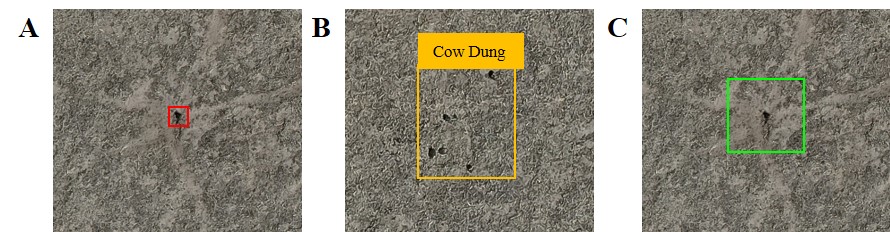


**Supplementary Figure 5.** Two kinds of labeling methods. (A) labeled using Label–1 method, (B) distractors, and (C) labeled using Label–2 method


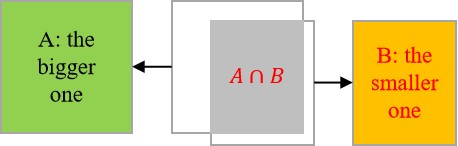


**Supplementary Figure 6.** Schematic diagram of the IoS.

# Supplementary Tables

**Supplementary Table 1** The hyperparameters of different model training.

| **Models** | **Input shape** | **Batch size** | **Learning rate** | **Weight decay** | **Optimizer** |
| --- | --- | --- | --- | --- | --- |
| Faster–rcnn | [600,600] | 8 | 0.0001 | 0 | Adam |
| SSD | [300,300] | 32 | 0.002 | 0.0005 | SGD |
| FCOS | [640,640] | 16 | 0.0003 | 0 | Adam |
| YOLOv3/YOLOv4 | [416,416] | 32 | 0.01 | 0.0005 | SGD |
| YOLOX/YOLOv5 | [640,640] | 32 | 0.01 | 0.0005 | SGD |
| YOLOv7 | [640,640] | 16 | 0.01 | 0.0005 | SGD |
